# Clinical characteristics and healthcare utilization of orthostatic dysregulation in Japan: a nationwide descriptive study

**DOI:** 10.1186/s13030-026-00363-1

**Published:** 2026-06-25

**Authors:** Kazuma Shinno, Hiroyuki Iijima, Kazue Ishitsuka, Norihiko Inoue, Yuki Hashimoto, Akira Nagai, Isamu Kamimaki, Shinichiro Nagamitsu

**Affiliations:** 1 Clinical Research Center, National Hospital Organization (NHO) Saitama Hospital, Saitama, Japan; 2 Center for Postgraduate Education and Training, National Center for Child Health and Development (NCCHD), 2-10-1 Okura, Setagaya-ku, Tokyo, 157-8535 Japan; 3 Department of Social Medicine, NCCHD, Tokyo, Japan; 4 Department of General Pediatrics and Interdisciplinary Medicine, NCCHD, Tokyo, Japan; 5 Department of Life Course Epidemiology, Women’s Center, Integrated Center for Women and Health, NCCHD, Tokyo, Japan; 6 Department of Clinical Data Management and Research, Clinical Research Center, National Hospital Organization Headquarters, Tokyo, Japan; 7 Institute of Clinical Epidemiology, Showa Medical University, Tokyo, Japan; 8 Department of Health Policy and Informatics, Graduate School of Medical and Dental Sciences, Institute of Science Tokyo, Tokyo, Japan; 9 Department of Pediatrics, NHO Saitama Hospital, Saitama, Japan; 10 Department of Pediatrics, Fukuoka University, Fukuoka, Japan

**Keywords:** Descriptive epidemiology, Healthcare utilization, Nationwide survey, Orthostatic dysregulation, Psychosomatic disorders, School non-attendance

## Abstract

**Background:**

Orthostatic dysregulation (OD) is a physical disorder characterized by circulatory disturbances due to autonomic nervous system imbalance that predominantly affects children. In Japan, OD is common and closely associated with school non-attendance, and its clinical management has developed from a psychosomatic perspective. However, large-scale data on real-world clinical practices are lacking. This study uses nationwide data to describe the comorbidities, prescribed medications, and medical practices for OD.

**Methods:**

This descriptive study utilized the Medical Information Analysis databank, a nationwide administrative database operated by the National Hospital Organization in Japan. We identified 7,213 patients aged 6 to 18 years who were first diagnosed with OD between 2016 and 2023. Descriptive statistics were used to analyze patient demographics, comorbidities, prescribed medications, and medical practices. Subgroup analyses stratified by prescription patterns were conducted.

**Results:**

The median age at diagnosis was 13 years, and 55.6% of the patients were female. The most common comorbidity category was mental health/behavioral disorders (23.6%). The most prevalent specific conditions were sleep disorders (10.2%), school non-attendance (9.3%), and autism spectrum disorder/attention-deficit hyperactivity (7.1%). Pharmacotherapy was provided to 44.0% of the patients, with a mean of 1.3 medications per patient. Midodrine was the most prescribed medication (78.3%), followed by amezinium metilsulfate (6.7%), and propranolol (2.8%). Sleep medications were prescribed to 9.4% of treated patients. The most frequent medical practices were blood tests (48.6%), diagnostic imaging (35.9%), and electrocardiograms (24.2%). The subgroup prescribed sleep medications had a higher prevalence of neurodevelopmental disorders, school non-attendance, and depression.

**Conclusions:**

This first nationwide descriptive study of OD in Japan provides real-world evidence on clinical characteristics and healthcare utilization. Heterogeneous comorbidity profiles and prescription patterns reveal the clinical diversity of the patient population. Our findings highlight the need for tailored therapeutic strategies to support more effective care.

**Supplementary Information:**

The online version contains supplementary material available at https://doi.org/10.1186/s13030-026-00363-1.

## Background

Orthostatic dysregulation (OD) is characterized by an inability to tolerate upright posture due to autonomic imbalance. Patients present with symptoms such as syncope, dizziness, and fatigue that impair quality of life [1, 2]. In Japan, OD is estimated to affect 5% of elementary school and 10% of junior and senior high school students [3].

Internationally, clinical presentations similar to OD are typically classified and managed as cardiovascular disorders, including orthostatic intolerance and postural orthostatic tachycardia syndrome (POTS). These conditions share overlapping symptoms with OD, such as dizziness, syncope, and exercise intolerance [4]. To date, a large-scale epidemiological study in China has found a POTS prevalence of 6.8% among adolescents, similar to the trend observed in Japan [5]. By contrast, the prevalence estimates range from 0.2% to 1.0% in Western countries [6, 7]. In the Japanese clinical context, while OD is recognized as a physical disorder, clinical management has evolved to incorporate a psychosomatic framework. This perspective reflects the fact that OD is a leading cause of prolonged school non-attendance in Japan, accounting for an estimated 30% to 40% of cases [4, 8]. The Ministry of Education, Culture, Sports, Science, and Technology in Japan reports that there are more than 350,000 students with school non-attendance [9]. Moreover, students with school non-attendance are likely to have somatic symptoms [10, 11].

OD represents an important clinical disorder that affects a large number of patients and impairing both quality of life and social functioning [8]. However, comprehensive data on the real-world management of OD, including comorbidities and medical resource utilization, remain limited. Previous studies in Japan have largely been restricted to single-center reports or surveys conducted at specialized psychosomatic medicine institutions [12]. Consequently, an evidence gap persists between general pediatric settings and specialized psychosomatic medicine institutions, highlighting the need for large-scale, nationwide epidemiological studies [13].

OD is widely recognized as a heterogeneous condition [2, 4, 6, 7]. While previous studies have reported associations with comorbidities such as sleep and psychiatric disorders [14, 15], to our knowledge, no study has yet attempted to statistically characterize these distinct clinical subgroups. We hypothesized that stratifying patients by prescription patterns could help characterize distinct clinical subgroups.

The aim of this study was to clarify the clinical characteristics and healthcare utilization of pediatric patients with OD in Japan using a real-world, nationwide, multicenter database, with a view to improving clinical management.

## Methods

### Study design and data source

This large-scale descriptive study utilized the Medical Information Analysis (MIA) databank, a nationwide administrative claims database operated by the National Hospital Organization (NHO) in Japan. The MIA database has collected daily administrative claims data from 140 hospitals affiliated with the National Hospital Organization since April 2010 and contains the records of over eight million patients [13]. The database includes patient demographics, diagnoses, medical procedures, medications, laboratory tests, and admission/discharge details. Diagnoses are recorded using the International Classification of Diseases, 10th Revision (ICD-10) codes, the Japanese insurance claim codes, and specific disease names, accompanied by the date of onset. Prescriptions and medical procedures are indexed by date and identified by National Health Insurance (NHI) Drug Price List codes and Japanese-specific claims codes, respectively. The validity of this database for clinical epidemiological research has been confirmed in previous studies [13]. The study period for this research was from January 2016 to March 2023.

### Study population

During the study period, approximately 578,000 patients aged 6 to 18 years were registered in the MIA database. We identified patients aged 6 to 18 years who were newly diagnosed with OD at inpatient or outpatient visits between January 2016 and March 2023. OD lacks a specific ICD-10 code; therefore, a two-step process was employed for patient identification. First, patients were screened using Japanese claims codes corresponding to OD and its recognized subtypes (listed in Supplementary Material 1). Second, a string-matching algorithm was applied to the free-text diagnostic labels recorded in Japanese.

This algorithm searched for disease name strings that matched established Japanese clinical terminology for OD subtypes, thereby reducing potential misclassification caused by inaccurate coding. Patients with diagnostic flags indicating “recurrence,” “exacerbation,” “history of,” or “suspected” were excluded from the analysis. Demographic data were then collected for the eligible population. The extraction codes for OD and its subtypes are provided in Supplementary Material 1.

### Variables and outcomes

We assessed patient demographics, comorbidities, prescribed medications, and medical practices.

Comorbidities were identified using ICD-10 codes (except for school non-attendance, which was identified using Japanese claims codes) recorded from one year before to three months after the initial OD diagnosis. The one-year pre-diagnostic window was chosen to capture comorbidities likely to precede or co-occur with OD onset, while the three-month post-diagnostic window was selected to reflect the early clinical course following diagnosis. Comorbidities were classified into major categories, including mental health/behavioral, neurological, and gastroenterological disorders. Specific conditions of interest encompassed school non-attendance, depression, sleep disorders, and autism spectrum disorder/attention-deficit hyperactivity disorder (ASD/ADHD). A detailed list of comorbidity categories and specific conditions is provided in Supplementary Material 1.

Medications and medical practices were examined if they were recorded in the month of or the month following the OD diagnosis. The one-month window was intended to capture initial therapeutic decisions at the time of OD diagnosis. Medications were identified by NHI Drug Price List codes. The medication list was curated based on clinical guidelines and literature reviews [2, 7, 16–18]. The medications prescribed were categorized as common OD medications (midodrine, amezinium metilsulfate, and propranolol) [3] or sleep medications (melatonin and ramelteon).

Medical practices were identified using Japanese reimbursement codes. The list of practices was selected from Japanese clinical guidelines for OD, focusing on procedures used to establish a diagnosis of OD and to rule out other organic conditions [3]. Laboratory tests included blood tests, urinalysis, and fecal occult blood tests. Physiological tests included electrocardiograms, echocardiograms, electroencephalograms, and head-up tilt tests. Active stand tests were excluded because they lack a specific reimbursement code.

### Statistical analysis

Descriptive data were summarized as medians and interquartile ranges (IQRs) for continuous variables and as numbers and percentages for categorical variables. Patient demographics, comorbidities, prescriptions, and medical practices were compared across prescription-based subgroups using Pearson’s chi-square test. A P-value < 0.05 was regarded as statistically significant. To supplement P-values, risk differences with 95% confidence intervals (CIs) were calculated for subgroup comparisons. Because these analyses were exploratory and descriptive, no formal correction for multiple testing was applied. Annual numbers of incident OD diagnoses were tabulated by the calendar year of recorded disease onset and compared between the pre-COVID-19 (2016–2019) and post-COVID-19 (2020–2022) periods using the Wilcoxon rank-sum test (Supplementary Material 2). All statistical analyses were performed using R version 4.3.0 [19].

### Ethics considerations

This study adhered to the Declaration of Helsinki and the Ethics Guidelines for Clinical/Epidemiological Studies of the Japanese Ministry of Health, Labor, and Welfare, and approval was obtained from the ethics committee of the NHO Saitama Hospital, Japan (No. R 2024-04). Individual informed consent was waived due to the retrospective use of anonymized data.

### Declaration of generative AI and AI-assisted technologies in the writing process

While preparing this paper, the authors used Gemini (Gemini 3 pro, by Google) to enhance the readability and proofread the initial English draft. After using this tool, the authors reviewed and edited the content as needed and take full responsibility for the content of the publication.

## Results

### Patient characteristics

The data of 7,213 patients from 122 hospitals were included in the study. The median age at diagnosis was 13 years (IQR, 12–14), and 55.6% of the patients were female (Table 1). The annual number of incident OD diagnoses generally increased over the study period, with a decline in 2020 followed by a recovery in 2021–2022. The mean annual count did not differ significantly between the pre-COVID-19 and post-COVID-19 periods (Wilcoxon rank-sum test, *P* = 0.23; Supplementary Material 2).

**Table 1:** Patient demographics and comorbidities of orthostatic dysregulation

	Total patient	Subgroup prescribed	Subgroup without prescription	*p*-value^2^	Difference % (95% CI)	Subgroup prescribed sleep medications	Subgroup not prescribed sleep medications	*p*-value^2^	Difference % (95% CI)
Patient, n	7,213	3,172	4,041			297	2,875		
Age, Median [IQR(25%-75%)]	13 [12–14]	13 [12–14]	13 [12–14]			14 [13–14]	13 [12–14]		
Sex, Female^1^	4,011 (55.6%)	1,794 (56.6%)	2,217 (54.9%)			167 (56.2%)	1,627 (56.6%)		
Comorbidity category^1^									
Metabolic/Endocrine disorders	1,253 (17.4%)	644 (20.3%)	609 (15.1%)	< 0.001	5.2 (3.5 to 7.0)	46 (15.5%)	598 (20.8%)	0.030	-5.3 (-9.7 to -0.9)
Mental health/behavioural disorders	1,702 (23.6%)	725 (22.9%)	977 (24.2%)	0.190	-1.3 (-3.3 to 0.7)	120 (40.4%)	605 (21.0%)	< 0.001	19.4 (13.6 to 25.1)
Neurological disorders	574 (8.0%)	306 (9.6%)	268 (6.6%)	< 0.001	3.0 (1.7 to 4.3)	26 (8.8%)	280 (9.7%)	0.584	-1.0 (-4.4 to 2.4)
Respiratory disorders (except asthma)	114 (1.6%)	61 (1.9%)	53 (1.3%)	0.039	0.6 (0.0 to 1.2)	6 (2.0%)	55 (1.9%)	0.898	0.1 (-1.6 to 1.8)
Cardiovascular disorders	17 (0.2%)	9 (0.3%)	8 (0.2%)	0.456	0.1 (-0.1 to 0.3)	0 (0.0%)	9 (0.3%)	NA	-0.3 (-0.5 to -0.1)
Hematological/Immunological disorders	431 (6.0%)	219 (6.9%)	212 (5.2%)	0.003	1.7 (0.5 to 2.8)	19 (6.4%)	200 (7.0%)	0.717	-0.6 (-3.5 to 2.4)
Gastroenterological disorders	1,271 (17.6%)	768 (24.2%)	503 (12.4%)	< 0.001	11.8 (10.0 to 13.6)	74 (24.9%)	694 (24.1%)	0.766	0.8 (-4.4 to 5.9)
Specific comorbidities^1^									
School non-attendance	671 (9.3%)	258 (8.1%)	413 (10.2%)	0.002	-2.1 (-3.4 to -0.8)	54 (18.2%)	204 (7.1%)	< 0.001	11.1 (6.6 to 15.6)
Depression	198 (2.7%)	81 (2.6%)	117 (2.9%)	0.378	-0.3 (-1.1 to 0.4)	17 (5.7%)	64 (2.2%)	< 0.001	3.5 (0.8 to 6.2)
Sleep disorder	734 (10.2%)	457 (14.4%)	277 (6.9%)	< 0.001	7.6 (6.1 to 9.0)	269 (90.6%)	188 (6.5%)	< 0.001	84.0 (80.6 to 87.5)
Autism spectrum disorder/Attention-deficit hyperactivity disorder	515 (7.1%)	177 (5.6%)	338 (8.4%)	< 0.001	-2.8 (-4.0 to -1.6)	59 (19.9%)	118 (4.1%)	< 0.001	15.8 (11.2 to 20.4)
Irritable bowel syndrome	477 (6.6%)	292 (9.2%)	185 (4.6%)	< 0.001	4.6 (3.4 to 5.8)	28 (9.4%)	264 (9.2%)	0.889	0.3 (-3.2 to 3.7)
Asthma	359 (5.0%)	213 (6.7%)	146 (3.6%)	< 0.001	3.1 (2.1 to 4.2)	20 (6.7%)	193 (6.7%)	0.989	0.0 (-3.0 to 3.0)
Eating disorder	42 (0.6%)	22 (0.7%)	20 (0.5%)	0.271	0.2 (-0.2 to 0.6)	4 (1.3%)	18 (0.6%)	0.144	0.7 (-0.6 to 2.1)
Iron-deficiency anemia	370 (5.1%)	187 (5.9%)	183 (4.5%)	0.009	1.4 (0.3 to 2.4)	17 (5.7%)	170 (5.9%)	0.895	-0.2 (-3.0 to 2.6)

(1) n (%), (2) Pearson’s Chi-squared test　Abbreviation: interquartile range: IQR; 95% confidence interval: 95% CI; Not available: NA

### Prevalence of comorbidities

The most common comorbidity categories were mental health/behavioral disorders (23.6%), gastroenterological disorders (17.6%), and metabolic/endocrine disorders (17.4%). Key specific comorbidities included sleep disorders (10.2%), school non-attendance (9.3%), autism spectrum disorder/attention-deficit hyperactivity disorder (ASD/ADHD) (7.1%), and depression (2.7%). The subgroup receiving any prescription had a higher prevalence of systemic comorbidity categories, including metabolic/endocrine, neurological, respiratory, hematological/immunological, and gastroenterological disorders. They also showed a higher prevalence of specific comorbidities, including sleep disorder, irritable bowel syndrome, asthma, and iron-deficiency anemia. The subgroup prescribed sleep medications showed a higher prevalence of mental health/behavioral and metabolic/endocrine disorders. This subgroup frequently presented with school non-attendance, depression, sleep disorder, and ASD/ADHD (Table 1).

### Prescribed medications

Overall, 44.0% of the patients received at least one prescription, with a mean of 1.3 medications per patient. Among the treated subgroup, 83.6% were prescribed medications commonly used for OD. Midodrine was the most frequently prescribed medication (78.3%), followed by amezinium metilsulfate (6.7%) and propranolol (2.8%). Sleep medications were prescribed to 9.4% of treated patients, with ramelteon used in 7.5% and melatonin in 1.9%. Various types of Japanese herbal medicines (Kampo) were also prescribed. Subgroup analysis revealed that the subgroup not prescribed common OD medications were significantly more likely to receive sleep medications (*P* < 0.001) and specific Kampo medicines (Goreisan, Shokenchuto, Hangebyakujutsutemmato, Hochuekkito, and Ryokeijutsukanto; *P* < 0.001 for each). Conversely, patients in the subgroup prescribed sleep medications were significantly less likely to receive common OD medications (*P* < 0.001) (Table 2).

**Table 2:** Distribution of prescriptions among patients with orthostatic dysregulation

		Subgroup prescribed	Subgroup prescribed any medication for orthostatic dysregulation	Subgroup not prescribed medications for orthostatic dysregulation	Subgroup prescribed sleep medications	Subgroup not prescribed sleep medications
Patient, n		3,172	2,653	519	297	2,875
Medications	Medication classes					
Midodrine	Adrenergic and dopaminergic agonists	2,483 (78.3%)	2,483 (93.6%)	0 (0.0%)	194 (65.3%)	2,289 (79.6%)
Amezinium metilsulfate	Adrenergic and dopaminergic agonists	212 (6.7%)	212 (8.0%)	0 (0.0%)	13 (4.4%)	199 (6.9%)
Droxidopa	Adrenergic and dopaminergic agonists	3 (0.1%)	2 (0.1%)	1 (0.2%)	2 (0.7%)	1 (0.0%)
Propranolol	Beta-blockers	88 (2.8%)	88 (3.3%)	0 (0.0%)	10 (3.4%)	78 (2.7%)
Atenolol	Beta-blockers	2 (0.1%)	0 (0.0%)	2 (0.4%)	0 (0.0%)	2 (0.1%)
Bisoprolol fumarate	Beta-blockers	12 (0.4%)	6 (0.2%)	6 (1.2%)	3 (1.0%)	9 (0.3%)
Ivabradine	HCN channel blockers	9 (0.3%)	5 (0.2%)	4 (0.8%)	3 (1.0%)	6 (0.2%)
Desmopressin	Vasopressin analogs	7 (0.2%)	2 (0.1%)	5 (1.0%)	0 (0.0%)	7 (0.2%)
Fludrocortisone	Mineralocorticoids	1 (0.0%)	1 (0.0%)	0 (0.0%)	0 (0.0%)	1 (0.0%)
Melatonin	Melatonin receptor agonists	60 (1.9%)	43 (1.6%)	17 (3.3%)	60 (20.2%)	0 (0.0%)
Ramelteon	Melatonin receptor agonists	239 (7.5%)	164 (6.2%)	75 (14.5%)	239 (80.5%)	0 (0.0%)
Tofisopam	Benzodiazepines	21 (0.7%)	11 (0.4%)	10 (1.9%)	2 (0.7%)	19 (0.7%)
Zolpidem tartrate	Benzodiazepines	14 (0.4%)	10 (0.4%)	4 (0.8%)	3 (1.0%)	11 (0.4%)
Tandospirone citrate	Serotonergic anxiolytics	34 (1.1%)	19 (0.7%)	15 (2.9%)	6 (2.0%)	28 (1.0%)
Pyridostigmine	Cholinesterase inhibitors	1 (0.0%)	1 (0.0%)	0 (0.0%)	1 (0.3%)	0 (0.0%)
Mecobalamin	Vitamins	17 (0.5%)	8 (0.3%)	9 (1.7%)	4 (1.3%)	13 (0.5%)
Goreisan	Kampo medicine	124 (3.9%)	80 (3.0%)	44 (8.5%)	10 (3.4%)	114 (4.0%)
Shokenchuto	Kampo medicine	141 (4.4%)	91 (3.4%)	50 (9.6%)	6 (2.0%)	135 (4.7%)
Shinbuto	Kampo medicine	4 (0.1%)	2 (0.1%)	2 (0.4%)	0 (0.0%)	4 (0.1%)
Hangebyakujutsutemmato	Kampo medicine	149 (4.7%)	86 (3.2%)	63 (12.1%)	5 (1.7%)	144 (5.0%)
Hochuekkito	Kampo medicine	126 (4.0%)	88 (3.3%)	38 (7.3%)	17 (5.7%)	109 (3.8%)
Ryokeijutsukanto	Kampo medicine	112 (3.5%)	55 (2.1%)	57 (11.0%)	9 (3.0%)	103 (3.6%)
Sleep medications		297 (9.4%)	205 (7.7%)	92 (17.7%)	297 (100.0%)	0 (0.0%)
Medications for orthostatic dysregulation		2,653 (83.6%)	2,653 (100.0%)	0 (0.0%)	205 (69.0%)	2,448 (85.1%)
Number of medications						
1		2,371 (74.7%)	1,911 (72.0%)	460 (88.6%)	73 (24.6%)	2,298 (79.9%)
2		651 (20.5%)	601 (22.7%)	50 (9.6%)	162 (54.5%)	489 (17.0%)
3		120 (3.8%)	111 (4.2%)	9 (1.7%)	49 (16.5%)	71 (2.5%)
≥ 4		30 (0.9%)	30 (1.1%)	0 (0.0%)	13 (4.4%)	17 (0.6%)
Mean number of medications (SD)		1.3 (0.6)	1.3 (0.6)	1.1 (0.4)	2.0 (0.8)	1.2 (0.5)

Footnote: Sleep medications included melatonin or ramelteon. Medications for orthostatic dysregulation (OD) included midodrine, amezinium metilsulfate, or propranolol. Although metoprolol, clonidine, Saikokeishito, and Ogikenchuto may be used for the treatment of OD according to expert opinion, no patients were treated with these medications in this study. Abbreviation: standard deviation: SD

### Medical practices

The most frequently performed medical practices were blood tests (48.6%), diagnostic imaging (35.9%), and electrocardiograms (24.2%). Overall, laboratory data were available for 49.1% of the patients, and physiological tests were performed for 29.3%. The subgroup receiving any prescription generally underwent medical practices at higher rates. Subgroup analysis indicated that the subgroup prescribed sleep medications generally underwent medical practices less frequently. In contrast, specialized psychiatric therapy (Difference: 14.9%; 95% CI: 10.2–19.6; *P* < 0.001) and medical guidance and management fees (Difference: 9.1%; 95% CI: 4.6–13.6; *P* < 0.001) were significantly more common in this subgroup (Table 3).

**Table 3:** Medical intervention for patients with orthostatic dysregulation

	Total patient	Subgroup prescribed	Subgroup without prescription	*p*-value*	Difference % (95% CI)	Subgroup prescribed sleep medications	Subgroup not prescribed sleep medications	*p*-value*	Difference % (95% CI)
Patient, n	7,213	3,172	4,041			297	2,875		
Laboratory data	3,543 (49.1%)	1,964 (61.9%)	1,579 (39.1%)	< 0.001	22.8 (20.6 to 25.1)	166 (55.9%)	1,798 (62.5%)	0.025	-6.7 (-12.6 to -0.7)
Blood test	3,503 (48.6%)	1,938 (61.1%)	1,565 (38.7%)	< 0.001	22.4 (20.1 to 24.6)	165 (55.6%)	1,773 (61.7%)	0.040	-6.1 (-12.0 to -0.2)
Urinalysis	1,268 (17.6%)	732 (23.1%)	536 (13.3%)	< 0.001	9.8 (8.0 to 11.6)	64 (21.5%)	668 (23.2%)	0.511	-1.7 (-6.6 to 3.2)
Fecal occult blood test	112 (1.6%)	69 (2.2%)	43 (1.1%)	< 0.001	1.1 (0.5 to 1.7)	0 (0.0%)	69 (2.4%)	NA	-2.4 (-3.0 to -1.8)
Physiological tests	2,111 (29.3%)	1,005 (31.7%)	1,106 (27.4%)	< 0.001	4.3 (2.2 to 6.4)	68 (22.9%)	937 (32.6%)	< 0.001	-9.7 (-14.8 to -4.6)
Electrocardiogram	1,745 (24.2%)	833 (26.3%)	912 (22.6%)	< 0.001	3.7 (1.7 to 5.7)	58 (19.5%)	775 (27.0%)	0.006	-7.4 (-12.2 to -2.6)
Echocardiography	443 (6.1%)	197 (6.2%)	246 (6.1%)	0.829	0.1 (-1.0 to 1.2)	17 (5.7%)	180 (6.3%)	0.715	-0.5 (-3.3 to 2.3)
Electroencephalogram	571 (7.9%)	259 (8.2%)	312 (7.7%)	0.488	0.4 (-0.8 to 1.7)	15 (5.1%)	244 (8.5%)	0.039	-3.4 (-6.1 to -0.8)
Head-up tilt test	57 (0.8%)	26 (0.8%)	31 (0.8%)	0.802	0.1 (-0.4 to 0.5)	4 (1.3%)	22 (0.8%)	0.298	0.6 (-0.8 to 1.9)
Diagnostic imaging	2,589 (35.9%)	1,430 (45.1%)	1,159 (28.7%)	< 0.001	16.4 (14.2 to 18.6)	98 (33.0%)	1,332 (46.3%)	< 0.001	-13.3 (-19.0 to -7.7)
Medical guidance and management fees	498 (6.9%)	326 (10.3%)	172 (4.3%)	< 0.001	6.0 (4.8 to 7.3)	55 (18.5%)	271 (9.4%)	< 0.001	9.1 (4.6 to 13.6)
Injection	637 (8.8%)	398 (12.5%)	239 (5.9%)	< 0.001	6.6 (5.3 to 8.0)	33 (11.1%)	365 (12.7%)	0.433	-1.6 (-5.4 to 2.2)
Specialized psychiatric therapy	584 (8.1%)	245 (7.7%)	339 (8.4%)	0.304	-0.7 (-1.9 to 0.6)	63 (21.2%)	182 (6.3%)	< 0.001	14.9 (10.2 to 19.6)

*Pearson’s Chi-squared test

Footnote: Physiological tests included electrocardiogram, echocardiography, electroencephalogram, and the head-up tilt test. Laboratory data included blood tests, urinalysis, and fecal occult blood tests

Abbreviation: 95% confidence interval: 95% CI; Not available: NA

## Discussion

In this study, we characterized the clinical features and real-world management of pediatric OD in Japan. Among more than 7,000 patients, 44.0% received pharmacological treatment, most commonly midodrine. Almost half of the patients underwent some medical interventions. Mental health and behavioral disorders were the most prevalent comorbidity categories, while sleep disorder, school non-attendance, and ASD/ADHD were also particularly frequent. Previous research has primarily been limited to single-center studies or surveys conducted at institutions specializing in psychosomatic medicine, thus may lack generalizability [12, 15, 20]. By leveraging a large-scale, nationwide administrative database, our study addresses this evidence gap and provides a comprehensive overview of the comorbidities, prescribed medications, and medical practices associated with OD.

While pediatric psychosomatic conditions, including OD, have been reported to increased following the COVID-19 pandemic [12], we did not observe a significant difference in the annual number of incident OD diagnoses between the pre- and post-COVID-19 periods. The transient decline in 2020 may reflect reduced healthcare access during the early phase of the pandemic, followed by a recovery thereafter.

The profile of comorbidities observed in this study is generally consistent with previous reports, characterized by high frequencies of sleep disorders, school non-attendance, ASD/ADHD, and depression. However, the overall prevalence of these comorbidities in our study population was lower than that reported in previous studies [4, 14, 15, 21–25]. A single-center study in the United States reported that 74% of pediatric POTs patients had moderate-to-severe anxiety, depression, or both [23]. Similarly, a study on pediatric POTS patients at a university hospital revealed that 14.3–30.6% of them scored above the cutoff for autism spectrum disorder traits [25]. This difference is likely attributable to variations in the study population. Unlike previous single-center studies from specialized psychosomatic institutions that handle severe or complex cases, our findings suggest that comorbidities in the general OD population across secondary and tertiary care are less prevalent than previously assumed.

Our results provide a nationwide overview of the pharmacological management of OD, revealing that more than half of the patients did not receive pharmacotherapy at diagnosis, reflecting adherence to guidelines prioritizing non-pharmacological care [3, 4]. Among the medications, midodrine was the most frequently prescribed (78.3%), followed by amezinium metilsulfate and propranolol, consistent with previous reports that identified midodrine as the first-line pharmacotherapy for OD [4, 20]. Furthermore, the use of various Kampo medicines suggests that clinicians may adjust treatments to individual patient presentations. Of the treated subgroup, 74.7% were managed with a single medication, indicating that polypharmacy is uncommon at the initial phase of treatment. These findings demonstrate that the pharmacological approach to OD in Japan generally aligns with clinical guidelines, while also being tailored to individual clinical presentations.

Notably, we observed that the subgroup prescribed sleep medications (9.4% of those prescribed) had a significantly high prevalence of ASD/ADHD, school non-attendance, and depression. This observation is consistent with previous studies describing the co-occurrence of autonomic dysfunction, sleep disorders, and neurodevelopmental traits. One study noted a high prevalence of OD (70%) in adolescents with circadian rhythm sleep disorders [14]. Moreover, another study reported that autonomic dysfunction is over-represented in patients with ASD [22]. These findings suggest that the OD population is heterogeneous. Patients requiring sleep medications likely represent a clinically distinct subgroup characterized by more complex backgrounds involving neurodevelopmental and psychiatric features. Although this stratification may reflect differences in clinical practice patterns or care pathways, the clinical relevance of these findings remains meaningful. Treatment focused solely on OD symptoms may be insufficient for this subgroup, necessitating tailored therapeutic strategies including sleep and mental health management.

This study found that a relatively high proportion of patients underwent diagnostic workups, with blood tests (48.6%), diagnostic imaging (35.9%), and electrocardiograms (24.2%) being the most common procedures. This suggests that clinicians actively screen for organic diseases to rule out other causes of the patients’ symptoms, a practice that aligns with clinical guideline recommendations [3]. In contrast, the rate of medical guidance and management fees (6.9%) was lower than that reported in the survey by the Japanese Society of Psychosomatic Pediatrics [12]. This discrepancy may be attributable to our short observation window (the first month after diagnosis): these fees might not be consistently billed within this initial period. Alternatively, there may be less awareness or utilization of psychosomatic management fees among general pediatricians compared to the specialists in the society’s study. Generally, medical intervention was more frequently done for the subgroup not prescribed sleep medications. This may indicate that when psychiatric comorbidities are less prominent, somatic complaints become the primary concern, prompting a more extensive workup for physical conditions.

The present study has several limitations. First, the MIA databank used in this study covers mainly secondary and tertiary care institutions [13]. Thus, our study population may represent a more complex patient population than seen in primary care settings [26], which may have led to an overestimation of comorbidity prevalence and medical resource utilization. Despite this limitation, the nationwide coverage of the MIA databank compensates for the limited availability of medical procedures and laboratory tests in primary care settings. Consequently, this study is well-suited to bridging the evidence gap in the clinical management of OD between general pediatric settings and specialized psychosomatic medicine institutions. Second, although NHO hospitals are distributed across Japan, facility-level information such as regional classification and hospital size was not available, precluding analysis of regional or institutional variation. Third, the reliance on administrative claims data may have contributed to lower prevalence rates of comorbidities. Clinicians may prioritize coding for the primary diagnosis (OD) for reimbursement purposes, while under-reporting co-existing psychiatric or neurodevelopmental conditions. Fourth, our observation window was limited to three months after the initial OD diagnosis, potentially leading to the under-recognition of conditions identified later in the clinical course. In particular, psychiatric or neurodevelopmental diagnoses may be coded later, leading to their prevalence being underestimated within this window. Finally, because OD is a clinical entity primarily recognized in Japan and lacks a specific ICD-10 code, the possibility of misclassification cannot be completely excluded. To enhance data reliability, we used the validated MIA databank and employed a two-step identification approach that combined claims code–based screening with a string-matching algorithm. This algorithm was applied to free-text diagnostic labels in Japanese, targeting disease name strings consistent with established OD terminology.

## Conclusion

This nationwide descriptive study of OD in Japan characterizes the prevalence of specific comorbidities and diverse patterns of pharmacotherapy and medical practices. Sleep disorders, school non-attendance, and neurodevelopmental conditions were frequently observed. Our findings highlight the clinical heterogeneity of OD. Specifically, the subgroup requiring sleep medications showed higher psychiatric and neurodevelopmental burdens, suggesting the need for a tailored approach. This research offers valuable insights into the real-world management of OD, which will enable clinicians to deliver more comprehensive and effective patient care.

## Supplementary Information

Below is the link to the electronic supplementary material.

Supplementary Material 1 : ICD-10 codes and disease codes for orthostatic dysregulation and comorbidities of patients. A detailed list of orthostatic dysregulation, comorbidity categories, and specific conditions encoded using the International Classification of Diseases, 10th Revision (ICD-10) and the Japanese claims codes.

Supplementary Material 2 : Annual number of incident orthostatic dysregulation (OD) diagnoses (2016–2022). Counts are based on the calendar year of recorded disease onset, which may differ from the date of the patient's visit used for study inclusion and may predate it. The study period spanned January 2016 to March 2023. To compare complete calendar years, the partial year 2023 (January-March; n = 244) and cases with a recorded onset before 2016 (n = 62) were excluded; the annual counts therefore sum to 6,907, which differs from the total study population (n = 7,213). The mean annual count was 922 (range 839-1,116) in the pre-COVID-19 period (2016-2019) and 1,073 (range 962-1,142) in the post-COVID-19 period (2020-2022); the two periods did not differ significantly (Wilcoxon rank-sum test, P = 0.23).

## Data Availability

The datasets generated or analyzed during this study are not publicly available because they contain sensitive personal information. The analytical code is available from the corresponding author upon request.

## Abbreviations

ASD/ADHD: Autism spectrum disorder/attention-deficit hyperactivity disorder
ICD-10: International Classification of Diseases,10th Revision
IQR: Interquartile range
MIA: Medical Information Analysis
NHI: National Health Insurance
NHO: National Hospital Organization
OD: Orthostatic dysregulation
POTS: Postural orthostatic tachycardia syndrome
95% CI: 95% confidence interval
