# Supplementary material for: Clinical characteristics and healthcare utilization of orthostatic dysregulation in Japan: a nationwide descriptive study
Source: Biopsychosoc Med. 2026 Jun 25;20:16. doi: 10.1186/s13030-026-00363-1 (PMC13563752; doi:10.1186/s13030-026-00363-1)
Supplement: Supplementary file 1 [file 13030_2026_363_MOESM1_ESM.docx]

Supplementary Material 1. ICD-10 codes and disease codes for orthostatic dysregulation and comorbidities of patients

|  | ICD-10 codes (disease codes) |
| --- | --- |
| **Orthostatic dysregulation** | orthostatic dysregulation: (3379008); postural orthostatic tachycardia syndrome: (8850681); vasovagal syncope: (8833093); delayed orthostatic hypotension: (8832713) and disease name; instantaneous orthostatic hypotension: disease name |
| **Comorbidity categories** |  |
| Metabolic/Endocrine disorders | E00-E07,E10-E14,E20-E35,D50-D53,E50-E64,E66,E67,E70-E90,O24,  G590,G632,Z863,G736,Y423,N083,M142,H280,H360,Z131,T383,Z833 |
| Mental health/behavioral disorders | F20-F29,F30-F39,F40-F48,F60-F69,F70-F79,F90-F98, F50,F54,F81,F82,F83,F84,F88,F89,F800,F801,F802,F808,F809 |
| Neurological disorders | G00-G09,G10-G14,G35-G37,G80-G83,G60-G64,Q00-Q07, G41,G70,G71,G90,G91,G92,G93,G94,G96,G98,P10,P52,P57,P90,Q90,Q91,Q92,  Q93,I60,I61,I62,I63,I64,I65,I66,I67,I69,F803,G400,G401,G402,G403,G404,  G406,G407,G408,G409,P911,P912,P916,P210,Q860,Q861,Q868,R568,Z982,  T850,T851,Y460,Y461,Y462,Y463,Y464,Y465,Y466 |
| Respiratory disorders (except asthma) | J60-J70,J80-J84,J85-J86,Q30-Q34,Q35-Q37,  J40,J41,J42,J43,J44,J47,J98,E84,P75, J961,Q790,Z430,Z930,Z942 |
| Cardiovascular disorders | Q20,Q21,Q22,Q23,Q24,Q25,Q26,Q28,Z95,Q893,Z941,Z450,T820,T821,T822,  T823,T825,T826,T827,T828,T829,T862,Y605,Y615,Y625 |
| Hematological/Immunological disorders | D70-D77,D50-D53, D58,D64,D66,D67,D69,D80,D81,D82,D83,D84,D86,D89,  D560,D561,D562,D564,D568,D569,D570,D571,D572,D578,D610,D619,D680,  D681,D682,D684,D685,D686,D687,D688,D689,M904,N082,Z862 |
| Gastroenterological disorders | Q38-Q45,K40-K46,K50-K52,P75-P78, K21,K22,K25,K26,K27,K28,K30,K31,K58,K59,K71,K73,K90,S36,S37,  K293,K294,K295,K551,K552,K601,K602,K603,K604,K605,K721,K811,  K861,K862,K863,K868,Z931,Z932,Z933,Z934,Z944,Z948,Z980,E841,P836,P921 |
| **Specific comorbidities** |  |
| School non-attendance | (8843956) |
| Depression | F32,F33,F92,F204,F251,F313,F314,F315,F412,F920 |
| Sleep disorder | F51,G47 |
| Autism spectrum disorder/Attention-deficit hyperactivity disorder | F80,F81,F82,F83,F84,F88,F89,F90 |
| Irritable bowel syndrome | K58 |
| Asthma | J45,J46,T486,Y556,Z825 |
| Eating disorder | F50 |
| Iron-deficiency anemia | D50 |
