# Supplementary material for: Clinical characteristics and healthcare utilization of orthostatic dysregulation in Japan: a nationwide descriptive study
Source: Biopsychosoc Med. 2026 Jun 25;20:16. doi: 10.1186/s13030-026-00363-1 (PMC13563752; doi:10.1186/s13030-026-00363-1)
Supplement: Supplementary file 2 [file 13030_2026_363_MOESM2_ESM.docx]

Supplementary Material 2. Annual number of incident orthostatic dysregulation (OD) diagnoses (2016–2022).

| Year | Number of incident OD diagnoses | Period |
| --- | --- | --- |
| 2016 | 842 | Pre-COVID-19 |
| 2017 | 839 | Pre-COVID-19 |
| 2018 | 891 | Pre-COVID-19 |
| 2019 | 1,116 | Pre-COVID-19 |
| 2020 | 962 | Post-COVID-19 |
| 2021 | 1,115 | Post-COVID-19 |
| 2022 | 1,142 | Post-COVID-19 |
